# Occupational stress and pregnancy-related hypertension and diabetes: Results from a nationwide prospective cohort

**DOI:** 10.5271/sjweh.4004

**Published:** 2022-03-31

**Authors:** Claudia Lissåker, Tomas Hemmingsson, Katarina Kjellberg, Petra Lindfors, Jenny Selander

**Affiliations:** 1 Unit of Occupational Medicine, Institute of Environmental Medicine, Karolinska Institutet, Stockholm, Sweden; 2 Department of Public Health Sciences, Stockholm University, Stockholm, Sweden; 3 Centre for Occupational and Environmental Medicine, Region Stockholm, Sweden; 4 Department of Psychology, Stockholm University, Stockholm, Sweden

**Keywords:** employment, gestational diabetes, gestational hypertension, preeclampsia, job exposure, job exposure matrix, psychosocial work environment, Sweden, workplace stress

## Abstract

**Objectives:**

Using a large, national, prospective cohort, while adjusting for other work exposures, this study aims to investigate whether exposure to occupational stress during pregnancy is associated with hypertensive disorders of pregnancy (HDP) and gestational diabetes.

**Methods:**

Our cohort consisted of 1 102 230 singleton births between 1994–2014 in Sweden, based on high-quality register data of Swedish pregnancies. Exposure to occupational stress was obtained from a job exposure matrix (JEM) constructed from 12 questions pertaining to the psychosocial work environment from the 1997–2013 cycles of Swedish Work Environment Survey, including approximately 75 000 individuals. We utilized the decision authority, demands, and social support indices. Decision authority and demands were combined to categorize occupations into low, active, passive, and high strain work. We estimated relative risks (RR) and adjusted for relevant confounders, such as age, smoking and other work exposures.

**Results:**

Occupations with lower levels of decision authority were associated with increased risks of 12–23% for HDP and preeclampsia and 36–58% for gestational diabetes compared to occupations with the highest levels of decision authority. Passive occupations had increased risks of 10% for HDP and preeclampsia and 15% for gestational diabetes when compared to low strain jobs. No significant associations were found for high strain occupations.

**Conclusions:**

As a whole, occupational stress was not consistently associated with pregnancy outcomes in our study. However, decision authority was associated with an increased risk for pregnancy-related complications. Further studies should investigate whether improvements in working conditions can help decrease these risks.

Hypertensive disorders of pregnancy (HDP), which includes gestational hypertension and preeclampsia, affect 5–10% of pregnant women (1) and accounts for an estimated 14% of maternal mortality worldwide (2), with high income countries having lower estimates than developing countries. Preeclampsia is the more severe form of HDP, affecting an estimated 2–5% of pregnancies around the world (3). HDP can result in negative birth outcomes, such as preterm and small-for-gestational-age birth (4). Another condition that occurs in pregnancy is gestational diabetes (GDM), which is thought to affect 0.6–15% of pregnancies and can also lead to similar adverse birth outcomes (5). Furthermore, studies have reported an increased risk of developing cardiovascular disease and type 2 diabetes (T2D) later in life among women who had HDP (4) and GDM (5). The causes of HDP and GDM are not well understood. However, the two conditions share some of the same risk factors, such as parity, maternal race/ethnicity, obesity, increased age, and insulin resistance (4, 5), indicating a related etiology.

Stress has been suggested as a potential factor in the development of HDP and GDM. One recent meta-analysis summarized the effects of general stress on pregnancy and reported an increased association between stress and both gestational hypertension and preeclampsia (6). Other studies have linked stress to glucose intolerance in pregnancy (7) as well as to GDM (8). However, one understudied source of stress in relation to pregnancy disorders is occupational.

One of the most well-studied methods of quantifying psychosocial working conditions is Karasek’s job demand–control (JDC) model (9). The JDC model attempts to characterize the psychosocial work environment through control and demands. Jobs with high levels of demands and low levels of control, so-called high strain jobs, are considered to have high levels of stress (9). The JDC model has been extensively used for a variety of outcomes and studies have shown that high strain jobs can have detrimental health effects, such as cardiovascular disease (10), obesity (11), and T2D (12).

However, few studies investigate how job demands and control affect HDP. Existing studies report positive associations (13–18), but only one reports statistically significant findings (15). However, these studies are limited by their small sample sizes, which leads to a very low number of exposed cases in certain categories and increased risk of chance findings. Furthermore, only one of these studies investigated the impact of the different dimensions included in the JDC model (18), and only two adjusted for other work exposures (14, 17). The exposures included were obtained by self-reports, which can be subject to variation and misclassification due to individuals’ perceptions. Furthermore, one study used retrospective reports (17), which can introduce recall bias. To our knowledge this is the first large-scale prospective study using register data to investigate the impact of occupational stress on HDP and the first to investigate its effects on GDM.

Using a large, national, prospective, register-based cohort, while adjusting for other work exposures, this study aims to investigate whether exposure to occupational stress during pregnancy is associated with HDP and GDM.

## Methods

### Data collection

To form the prospective cohort for this study, we obtained pregnancy data from the Swedish Medical Birth Register (MBR) for all births between 1994–2014. Data were recorded from visits to the prenatal care unit from week 10 of pregnancy until the birth of the child. Background characteristics collected in early pregnancy, such as mother’s age, occupation, nationality, smoking status, weight, height, parity, and diagnoses, are included in this register. We included women who had singleton pregnancies and reported working full- or part-time at the first prenatal care unit appointment, occurring around gestational week 10. We excluded women who were pregnant with more than one child (twins, triplets), reported not working, listed a non-working occupation (eg, homemaker or student), had an occupation listed that could not be coded, or had missing occupation. Of the 1 431 015 pregnancies included in the MBR listed as working at least part-time, 1 102 230 fulfilled the criteria and were included in this study and 1 080 850 had no missing covariate data.

Data on mother’s highest education at the time of delivery and marital status were obtained from the Longitudinal Integration Database for Health Insurance and Labor Market Studies (Swedish acronym LISA), covering all individuals aged ≥16, and were merged to MBR data utilizing the unique personal identification number given to Swedish residents.

### Exposure to occupational stress

To examine exposure to occupational stress, we used a job exposure matrix (JEM) for psychosocial workload. The JEM has been described elsewhere (19). Briefly, the JEM utilizes 12 items from the 1997–2013 cycles of the biennial Swedish Work Environment Survey, which sampled over 75 000 men and women. Response categories were scaled from 0–10 indicating the amount of time each person experienced the situation in question. For each item, an average response was created and divided into indices for each job category, separately for men and women, of which we only use measurements taken from women respondents. Details on the items included in each index are found in the supplementary material (www.sjweh.fi/article/4004). In this study, we utilized the decision authority, psychological demands, and social support indices. Each index was categorized according to their quartile distribution in the study population.

We created a variable to investigate job strain as proposed in the JDC model (9) with decision authority and demand split at the median. Those with the values above the median were considered to have high decision authority and/or demand, while those with levels below the median were considered to have low. These two variables were then combined to form four categories: low strain (high decision authority/low demand), active (high decision authority/high demand), passive (low decision authority/low demand), and high strain (low decision authority/high demand).

The JEM was linked to the cohort data using occupational codes. Each woman provided her occupational title during gestational week 10. An occupational hygienist recorded this as free text and then coded it according to the ISCO-88-based Swedish occupational classification 96 (SSYK96) coding system (20). SSYK96 is formatted in a 4-digit hierarchical level, with each digit, from left to right, providing more detail on the occupation. For most occupations, the 4-digit code was available and were matched to the JEM index values to which it corresponds. However, for a few (23 685 of 1 102 230), only the less-detailed 2- or 3-level SSYK96 code indicating occupational group was available and were merged to average measurements of the 4-digit JEM values.

### Outcome

Diagnosis codes came from the MBR and were reported at the time of delivery for each pregnancy using the International Classification of Diseases, ninth and tenth revisions (ICD-9 and ICD-10). In our data ICD-9 codes were found until 1996, after which ICD-10 was adopted. For HDP, we used ICD-9 codes ‘642’, ‘642D’, 642E’, ‘642F’, ‘642G’, and ‘642H’; and ICD-10 codes ‘O11’, ‘O13’, ‘O14.0’, ‘O14.1’, ‘O14.2’, ‘O149’, ‘O15.0’, ‘O15.1’, ‘O15.2’, and ‘O15.9’. To determine outcomes of preeclampsia only, we used ICD-9 ‘642E’, ‘642F’, and ‘642H’; and ICD-10 codes ‘O119’, ‘O14.0’, ‘O14.1’, ‘O14.2’, ‘O149’, ‘O15.0’, ‘O15.1’, ‘O15.2’, and ‘O15.9’. For GDM, we used ICD-9 codes ‘648A’ and ‘648W’, and ICD-10 codes ‘O24.4’ and ‘O24.9’.

### Potential confounders

We chose several confounders that have been shown to be associated with HDP and, by extension, preeclampsia and GDM (4, 21) as well as individual factors that can affect occupational stress (22, 23): maternal age at delivery (<25, 25–30, 30–35, or ≥35 years), smoking status at gestational week 10 (non-smokers, smokers), educational level (some high school or less, high school graduate, some university or higher), marital status (not in a registered partnership or living alone, married or in a registered partnership), family situation (living with the father, living alone or another arrangement), body mass index (BMI) calculated using height and weight [underweight (<18.5 kg/m^2^), normal weight (18.5–25 kg/m^2^), overweight (25–30 kg/m^2^), obese (≥30 kg/m^2^)], country of birth (Sweden, Europe excluding Sweden, and rest of the world), parity (primipara, multipara), and employment status (full-time, part-time).

In addition, we considered several occupational exposures obtained from other JEM: physical workload, noise, whole-body vibrations, and 46 different chemicals and particles, which were matched based on occupational code and year of exposure, where applicable. Scores of 8 different physical exposures from the physical workload JEM (24) (heavy lifting [≥15 kg], physically strenuous work, fast breathing due to physically strenuous work, forward bent position, twisted position, working with hands above shoulder level, repetitive work, and frequent bending and twisting) were summed and averaged to create the physical load index, which was then divided into quartiles. The noise JEM included information on annual average 8-hr occupational noise level in decibels [dB(A)] in five categories: <70, 70–74, 75–79, 80–84, and >85 dB(A) (25). Whole body vibration was categorized as 0–0.1, 0.1–0.3, 0.3–0.5, and >0.5 m/s^2^. Exposure to chemicals and particles were calculated by multiplying the proportion within an occupational group considered exposed by the estimated level exposed (26). All chemical and particles exposures were considered, but ultimately only aromatic hydrocarbon (ARHC) and chlorinated hydrocarbon (CHC) solvents and polycyclic aromatic hydrocarbons (PAH) were chosen as indicators for solvent and combustion-related exposures, respectively.

### Data analyses

All analyses were performed using SAS version 9.4 (SAS Institute, Cary, NC, USA). For each outcome, the confounders listed above were selected into the model based on their placement on a causal diagram. BMI was concluded to be part of the causal pathway; therefore it was excluded from the final model. In order to create a parsimonious model, we checked whether confounders changed the association between exposures and outcomes by ≥5%. Neither marital status nor family situation met this criteria and thus were left out of the models. No variables were added at this step. The final model consisted of the following: maternal age at delivery, smoking status, educational level, country of birth, parity, employment status, physical workload, noise, whole-body vibrations, and exposure to ARHC, CHC, and PAH.

For women with multiple births, we could not assume independence; therefore, we estimated relative risks (RR) using a modified Poisson regression for correlated binary data (27). Crude and adjusted models were created for each outcome (HDP, preeclampsia, GDM). Because some women who had a previous complication may change duties, go on leave, or reduce working hours, we conducted a sensitivity analysis by restricting the sample to women with a first-time pregnancy who reported full-time employment. A sensitivity analysis was done including year of birth and results remained the same.

This study was conducted with approval from the Stockholm ethical review board (no. 2018/1298-31/2).

## Results

Of 1 102 230 pregnancies included in this study, 1 080 850 had no missing information on covariates. Baseline characteristics are described in table 1. Women who reported working in jobs with lowest quartiles of decision authority and demands were more often <30 years old, smokers, reporting lower education, born outside of Sweden, and part-time workers than those in the highest quartile. The same pattern was seen for those working in jobs with a high level of support, with the exception that there were no differences in support between women who were born in Sweden or elsewhere.

**Table 1:** Comparison of baseline maternal characteristics between the highest and lowest quartiles (Q) of occupational exposure to three psychosocial stress domains during pregnancy [%].

	Total (%)	Occupational Psychosocial Stress					

		Decision authority		Demands		Support	
		
		Q1 (low) N=266 890	Q4 (high) N=285 078	Q1 (low) N=292 230	Q4 (high) N=283 399	Q1 (low) N=257 151	Q4 (high) N=280 634
Age (years)							
<25	120 662 (11.0)	13.5	4.4	17.6	3.0	4.8	12.1
25–30	360 194 (32.7)	34.1	25.3	36.1	27.5	26.8	34.7
30–35	402 572 (36.5)	34.2	44.1	30.6	43.5	42.3	35.7
≥35	218 802 (19.8)	18.2	26.2	15.7	26.0	26.1	17.5
Smoking							
Non-smokers	1 000 833 (90.8)	89.2	94.9	85.6	95.8	94.5	90.5
Smokers	86 006 (7.8)	9.3	3.7	12.9	2.8	4.1	8.1
Missing	15 391 (1.4)	1.5	1.4	1.5	1.4	1.4	1.4
Educational level							
Some high school or less	277 614 (25.2)	28.7	12.8	41.9	7.5	13.2	30.2
High school graduate	305 986 (27.7)	30.3	24.2	36.7	12.2	19.0	38.8
Some university or higher	513 550 (46.6)	40.6	62.6	20.7	79.9	67.3	30.6
Missing	5080 (0.5)	0.4	0.4	0.7	0.4	0.5	0.4
Country of birth							
Sweden	977 646 (88.7)	87.2	90.6	86.4	90.3	89.2	90.7
Europe (excl. Sweden)	63 549 (5.8)	6.2	5.3	6.8	5.6	6.0	5.2
Rest of the world	59 985 (5.4)	6.5	4.0	6.7	4.0	4.7	4.0
Missing	1050 (0.1)	0.1	0.1	0.1	0.1	0.1	0.1
Parity							
Primigravida	510 102 (46.3)	44.2	49.4	45.3	46.4	47.3	48.6
Multigravida	592 128 (53.7)	55.8	50.6	54.7	53.6	52.7	51.4
Employment status							
Full-time	719 330 (65.3)	59.3	76.9	58.0	73.6	74.5	68.4
Part-time	382 900 (34.7)	40.7	23.1	42.0	26.4	25.5	31.6

Results of the crude and adjusted analyses for all working women are shown in table 2. Compared to the highest level of decision authority, lower decision authority was associated with an increased risk of all three outcomes after adjusting for confounders. However, no dose–response patterns were indicated. The first quartile of decision authority was associated with a 1.12 greater risk (95% CI 1.08–1.15), the second quartile with a 1.23 greater risk (95% CI 1.19–1.27), and the third with a 1.13 greater risk (95% CI 1.09–1.16) of HDP than the highest quartile. Results were similar for preeclampsia. For GDM, women who reported working in occupations found in the first quartile of decision authority had a 1.36 greater risk (95% CI 1.28–1.46), the second quartile a 1.58 greater risk (95% CI 1.47–1.70), and the third a 1.37 greater risk (95% CI 1.28–1.47) than women who work in occupations with the highest levels of decision authority.

**Table 2:** Crude and adjusted ^a^ associations between psychosocial stress and hypertensive disorders of pregnancy (HDP), preeclampsia, and gestational diabetes, N=1 080 850. [RR=relative risk; CI=confidence interval; Q=quartile].

Occupational stress	HDP			Preeclampsia			Gestational Diabetes		
		
	Exposed Cases	Crude RR (95% CI)	Adjusted RR (95% CI)	Exposed Cases	Crude RR (95% CI)	Adjusted RR (95% CI)	Exposed Cases	Crude RR (95% CI)	Adjusted RR (95% CI)
Decision authority									
Q1 (low)	10 625	1.05 (1.03–1.08)	1.12 (1.08–1.15)	8215	1.11 (1.08–1.14)	1.15 (1.11–1.19)	2643	1.50 (1.41–1.59)	1.36 (1.28–1.46)
Q2	11 326	1.09 (1.07–1.12)	1.23 (1.19–1.27)	8725	1.15 (1.12–1.18)	1.24 (1.19–1.29)	2653	1.47 (1.38–1.55)	1.58 (1.47–1.70)
Q3	11 106	1.06 (1.03–1.09)	1.13 (1.09–1.16)	8574	1.12 (1.08–1.15)	1.15 (1.11–1.19)	2607	1.42 (1.34–1.51)	1.37 (1.28–1.47)
Q4 (high)	10 797	1.00	1.00	7923	1.00	1.00	1889	1.00	1.00
Demands									
Q1 (low)	11 709	1.06 (1.04–1.09)	1.06 (1.03–1.09)	9154	1.13 (1.09–1.16)	1.06 (1.02–1.10)	2997	1.43 (1.35–1.51)	1.16 (1.09–1.25)
Q2	10 879	1.12 (1.09–1.15)	1.07 (1.04–1.10)	8489	1.18 (1.15–1.22)	1.08 (1.04–1.12)	2740	1.48 (1.40–1.56)	1.18 (1.10–1.26)
Q3	10 555	1.03 (1.01–1.06)	0.98 (0.95–1.01)	7885	1.05 (1.02–1.08)	0.97 (0.94–1.01)	2008	1.03 (0.97–1.09)	0.98 (0.92–1.04)
Q4 (high)	10 711	1.00	1.00	7909	1.00	1.00	2047	1.00	1.00
Support									
Q1 (low)	9412	0.91 (0.88–0.93)	0.93 (0.90–0.96)	6974	0.86 (0.84–0.89)	0.92 (0.89–0.96)	1808	0.86 (0.81–0.91)	0.91 (0.85–0.98)
Q2	11 887	1.00 (0.97–1.02)	1.09 (1.06–1.12)	9041	0.97 (0.95–1.00)	1.08 (1.04–1.11)	2870	1.19 (1.12–1.25)	1.22 (1.15–1.30)
Q3	11 229	1.03 (1.01–1.06)	1.06 (1.02–1.09)	8595	1.02 (0.99–1.05)	1.04 (1.00–1.08)	2814	1.27 (1.21–1.35)	1.20 (1.13–1.29)
Q4 (high)	11 326	1.00	1.00	8827	1.00	1.00	2300	1.00	1.00

a Adjusted for: age, smoking, education, country of birth, parity, physical load, noise, whole-body vibrations, aromatic hydrocarbon solvents, chlorinated hydrocarbon solvents, and polycyclic aromatic hydrocarbons.

Working in occupations with the two lowest levels of demands (first and second quartile) were also associated with an increased risk of all three outcomes compared to the highest level of demands (fourth quartile). There was no clear increase in the risk of any of the outcomes associated with working in occupations falling in the third quartile of demands.

Decreasing levels of support showed a higher risk for all three outcomes, but only for the second and third quartiles, whereas the lowest quartile of support was associated with a statistically significant decrease in HDP, preeclampsia only, and GDM when compared to women who worked in occupations with the greatest level of support (table 2).

Table 3 shows the results when restricting the analyses to women who were in their first pregnancy and working full-time. Compared to the highest quartile of decision authority, all lower quartiles were associated with an increase in HDP, preeclampsia, and GDM. For demands, the second quartile was associated with an increased risk of all three outcomes. The lowest quartile of support continued to show a decreased risk for HDP, preeclampsia, and GDM. However, the second quartile of support showed an increased risk for GDM only.

**Table 3:** Crude and adjusted ^a^ associations between psychosocial stress and hypertensive disorders of pregnancy (HDP), preeclampsia, and gestational diabetes for first-time pregnancies with full-time employment, N=339 072. [RR=relative risk; CI=confidence interval; Q=quartile].

Occupational stress	HDP			Preeclampsia			Gestational Diabetes		
		
	Exposed cases	Crude RR (95% CI)	Adjusted RR (95% CI)	Exposed cases	Crude RR (95% CI)	Adjusted RR (95% CI)	Exposed cses	Crude RR (95% CI)	Adjusted RR (95% CI)
Decision authority									
Q1 (low)	4128	1.04 (1.00-1.09)	1.06 (1.02-1.11)	3314	1.10 (1.05-1.15)	1.10 (1.05-1.16)	552	1.31 (1.17-1.47)	1.33 (1.17-1.52)
Q2	4529	1.10 (1.06-1.14)	1.14 (1.09-1.20)	3604	1.15 (1.10-1.20)	1.14 (1.09-1.21)	562	1.28 (1.14-1.44)	1.40 (1.21-1.62)
Q3	4907	1.04 (1.00-1.08)	1.06 (1.01-1.10)	3897	1.08 (1.04-1.13)	1.07 (1.02-1.12)	636	1.27 (1.13-1.41)	1.28 (1.12-1.45)
Q4 (high)	5891	1.00	1.00	4489	1.00	1.00	626	1.00	1.00
Demands									
Q1 (low)	4506	1.05 (1.01-1.09)	1.04 (1.00-1.09)	3609	1.09 (1.04-1.14)	1.03 (0.98-1.09)	593	1.26 (1.12-1.41)	1.13 (0.98-1.29)
Q2	4146	1.11 (1.06-1.15)	1.07 (1.02-1.12)	3358	1.16 (1.11-1.22)	1.07 (1.02-1.13)	562	1.37 (1.22-1.53)	1.22 (1.07-1.39)
Q3	5321	1.00 (0.96-1.03)	0.97 (0.93-1.01)	4116	1.00 (0.96-1.04)	0.96 (0.92-1.00)	619	1.05 (0.94-1.18)	1.02 (0.91-1.15)
Q4 (high)	5482	1.00	1.00	4221	1.00	1.00	602	1.00	1.00
Support									
Q1 (low)	4807	0.92 (0.89-0.96)	0.91 (0.87-0.95)	3722	0.89 (0.85-0.93)	0.91 (0.87-0.96)	501	0.80 (0.71-0.90)	0.84 (0.73-0.96)
Q2	4878	1.01 (0.97-1.05)	1.04 (0.99-1.08)	3825	0.99 (0.95-1.03)	1.03 (0.98-1.08)	641	1.11 (1.00-1.24)	1.22 (1.08-1.38)
Q3	4272	1.01 (0.97-1.05)	1.00 (0.95-1.04)	3359	0.99 (0.95-1.04)	0.97 (0.92-1.02)	574	1.13 (1.01-1.27)	1.13 (0.99-1.28)
Q4 (high)	5498	1.00	1.00	4398	1.00	1.00	660	1.00	1.00

a Adjusted for: age, smoking, education, country of birth, physical load, noise, whole-body vibrations, aromatic hydrocarbon solvents, chlorinated hydrocarbon solvents, and polycyclic aromatic hydrocarbons.

Table 4 shows the results for the combination of decision authority and demands into a job strain variable. Those with active occupations had 0.93 lower risk (95% CI 0.90–0.96), and those with passive occupations a 1.10 higher risk (95% CI 1.07–1.14) of HDP compared to those with low strain jobs. A similar pattern was observed for preeclampsia. For GDM, working in an active occupation was associated with a 0.80 lower risk (95% CI 0.75–0.86), passive occupations were associated with a 1.15 increased risk (95% CI 1.07–1.23), and high strain occupations were associated with no increased risk when compared to low strain jobs. Effect sizes were similar in the analyses restricted to women pregnant for the first time and working full-time.

**Table 4:** Crude and adjusted ^a^ associations between job strain and hypertensive disorders of pregnancy (HDP), preeclampsia, and gestational diabetes for the full sample (N=1 080 850 ) and first-time pregnancies (N=339 072 ) with full-time employment [RR=relative risk; 95% CI=95% confidence interval].

Job strain ^b^	HDP			Preeclampsia			Gestational diabetes		
		
	Exposed cases	Crude RR (95% CI)	Adjusted RR (95% CI)	Exposed cases	Crude RR (95% CI)	Adjusted RR (95% CI)	Exposed cases	Crude RR (95% CI)	Adjusted RR (95% CI)
Full sample									
Low strain	8442	1.00	1.00	6514	1.00	1.00	2000	1.00	1.00
Active	10 986	0.98 (0.95–1.01)	0.93 (0.90–0.96)	8040	0.93 (0.90–0.96)	0.90 (0.87–0.94)	2007	0.76 (0.71–0.80)	0.80 (0.75–0.86)
Passive	14 146	1.08 (1.05–1.11)	1.10 (1.07–1.14)	11 129	1.10 (1.07–1.14)	1.10 (1.06–1.14)	3737	1.21 (1.14–1.27)	1.15 (1.07–1.23)
High strain	10 280	0.98 (0.95–1.00)	1.02 (0.99–1.05)	7754	0.95 (0.92–0.99)	1.02 (0.98–1.06)	2048	0.82 (0.77–0.87)	1.00 (0.94–1.07)
First-time pregnancies with full-time employment									
Low strain	4100	1.00	1.00	3249	1.00	1.00	536	1.00	1.00
Active	5768	0.95 (0.92–0.99)	0.93 (0.89–0.97)	4391	0.92 (0.88–0.96)	0.91 (0.86–0.96)	630	0.80 (0.71–0.89)	0.80 (0.70–0.92)
Passive	4552	1.08 (1.03–1.12)	1.08 (1.03–1.13)	3718	1.11 (1.06–1.16)	1.07 (1.01–1.13)	619	1.12 (1.00–1.26)	1.10 (0.96–1.27)
High strain	5035	0.98 (0.94–1.02)	1.00 (0.95–1.04)	3946	0.97 (0.92–1.01)	1.00 (0.95–1.06)	591	0.88 (0.78–0.99)	1.00 (0.88–1.14)

a Adjusted for: age, smoking, education, and country of birth, physical load, noise, whole-body vibrations, aromatic hydrocarbon solvents, chlorinated hydrocarbon solvents, and polycyclic aromatic hydrocarbons. Analyses on first-time pregnancies with full-time employment not adjusted for parity.

b Low strain=high decision authority and low demand; active=high decision authority and high demand; passive=low decision authority and low demand; high strain=low decision authority and high demand.

## Discussion

In our prospective, nationwide cohort, having an occupation with lower levels of decision authority was associated with approximately a 12–20% increase in the risk of HDP and preeclampsia and approximately a 35–60% increase in GDM compared to occupations with the highest levels of decision authority. No clear associations were found for occupational stress as a whole. Our results were corroborated by sensitivity analyses of the subsample of women in their first pregnancies working full-time. To our knowledge, this is the first large, prospective study to investigate the impact of occupational stress on these pregnancy outcomes while adjusting for a wide variety of occupational exposures.

A few smaller studies have focused on job stress and pregnancy-related hypertension and preeclampsia. Mostly, these studies use only JDC model and do not explore individual dimensions of job strain. One exception has found that jobs with moderate and low control were associated with a non-statistically significant increased risk for preeclampsia, but not gestational hypertension (18). We also found that lower demand was associated with an increased risk of all three outcomes, with the similar risks in the two lowest quartiles. In contrast, one previous study has investigated workload in connection to gestational hypertension and preeclampsia and found that for preeclampsia, both moderate and high workload were associated with increased odds, whereas for gestational hypertension only moderate was associated with increased odds when compared to low (18).

Of the studies that investigate job strain, only two report statistical significance (15, 16); however, all show that working in high strain jobs is associated with an increase in pregnancy-related hypertensive disorders (13–18). Of two studies investigating the impact of other types of job strain (15, 17) on preeclampsia and gestational hypertension, both find increased, but not statistically significant, risks of preeclampsia associated with passive and active work, as well as high strain, when compared to low strain work. The present study also finds an increased risk for both HDP and preeclampsia for passive jobs, but not for high strain jobs.

Although there are no previous studies on GDM and occupational stress, some studies have investigated its impact on diabetes. One pooled study of European countries has found that high strain jobs were associated with a 1.13 increased risk of T2D (12). Another Swedish study found that for middle-aged women, increased work demands were not associated with increased odds of T2D, and in fact, the middle category of work demands had non-statistically significant decreased odds (28). This study also found that low decision latitude jobs were associated with 2.4 times increased odds of T2D compared to high. GDM has been found to predict development of T2D later in life (5). Thus, it is plausible that low decision authority begins to affect glucose tolerance as early as pregnancy.

The differences in results from previous research may be due to differences in exposure measurement. In the present study, measurements do not come from individual-level exposures, which may affect the comparability to previous research. We also do not investigate the effects of skill discretion, since the questions from the survey that were regarded as skill discretion are included in the calculation of the physical load index, a variable we adjust for in the analysis. Decision authority has been used without skill discretion in a prior study, as it has been thought to be the more pertinent part of control in the context of the Swedish labor market (29). Moreover, it is possible that the demands explored in our study are so-called “challenge stressors” as opposed to “hindrance stressors,” the former of which is thought to be beneficial to personal growth and achievement at work (30), and may elicit different stress responses than the latter. In other words, jobs in the highest demand category can also offer opportunities that can increase satisfaction and commitment and mitigate the risk of stress. This may also explain why, our study found increased risks for passive work only (low control/ low demands). Similarly, we found a slightly protective effect of active work, which may be due to the combined protective effect of high demands and control, and no effect due to high job strain (low control, high demands).

We did not find a dose–response relationship for decision authority. Only one study was able to investigate dose–response and found that decreasing control was associated with increased, albeit non-statistically significant, risks of preeclampsia (18). Furthermore, one recent Swedish study using the JEM to explore depressive outcomes also found a lack of dose–response relationship for decision authority for women only (19). These similar results may be due to the types of occupations held by women in the lowest control category in the JEM. In Almroth et al (19), the lowest control category included some highly educated women, such as those in the educational and healthcare sectors, which can indicate that the risk associated with job control is potentially mitigated by having jobs with higher social status. Even though we do not see a dose–response, we still see an increased effect of lower levels of control on these outcomes.

The mechanism via which decreased control can affect the development of HDP and GDM is thought to be via physiological responses to stress. Long-term stress activates the hypothalamus-pituitary-adrenal axis, increasing the concentration of corticotropin-releasing hormones, which have been found to be increased in women with HDP (31), and cortisol, an insulin-antagonist that can contribute to insulin resistance (32). Stress can also increase pro-inflammatory cytokines, which are associated with pregnancy complications like preeclampsia and GDM (5, 33). Stress may result in poorer health behaviors during pregnancy, such as substance abuse and overeating, as well as affecting sleep quality (34). Lastly, working in occupations with low levels of control can prevent women from adapting their work tasks, schedule, and pace to their changing circumstances, and thus leading women to work when they are not feeling well and affecting their overall health and pregnancy.

Given this mechanism, we would also expect to see an increased risk with high strain occupations. This was not the case in our study. However, as we mentioned previously, high demands may elicit a positive stress response that mitigates the negative effects of low control. It is possible that for young women, having a demanding job that challenges one professionally, in a positive way, may be more important for well-being than the negative effects of the low control they may experience. This area should be further explored in future studies.

An extension of the JDC model suggests that the inclusion of social support in a three-way interaction can mitigate the detrimental effects of high strain jobs (35). However, in this study we decided to not test for the role social support on job strain as the simple JDC model has been more explored in the past and we found no clear patterns in our data to indicate that we should investigate further. From our results, it appears that having a low amount of support is associated with a small decrease in the risk for all three outcomes when compared to the highest support. One possibility is that women who have low support work in occupations that do not need any support, while conversely, higher levels of support are indicative of a decreased ability to meet job requirements, ie, support is provided because support is needed. Alternatively, for jobs characterized by the middle quartiles of support, where there is some evidence for increased risk of HDP and GDM, the extent or type of support provided may not be enough to mitigate detrimental effects on health.

This study has limitations. First, the exposure was not ascertained at the individual level nor was it specific to pregnant women only; therefore, there is possible misclassification. Individual-level ascertainment is unfeasible in a study of this size. The JEM was developed for general use and data were collected from a sample representing the general working population. Thus, misclassification between individuals is likely to be non-differential, which would attenuate the risk. Additionally, job exposure was determined around gestational week 10, which is early enough in the pregnancy that we believe psychosocial working conditions would be experienced in a similar manner to nonpregnant women. Second, we did not include information on leave and could not account for duration of exposure. All diagnoses were given at the end of the pregnancy, and we could not ensure that the leave taken preceded issues related to HDP or GDM. Similarly, we could not ensure that women who were classified as exposed were actively performing the job reported at gestational week 10 for the entirety of their pregnancy. Lastly, it is likely that some diagnoses such as gestational hypertension and GDM are underreported in the registers, which would result in our totals not reflecting all the cases among Swedish pregnant women between 1994 and 2014. However, all diagnostic codes used were routinely reported by a physician after the child was born, making it highly unlikely that assignment of ICD codes was associated with occupations, thus attenuating the results.

Some of the strengths of our study include its large sample and prospective design. Data came from a national register, which includes nearly complete data on approximately 99% of Swedish pregnancies (36), making this generalizable the entire Swedish population of pregnant women. Because outcomes are relatively rare, a large sample size is needed to obtain enough power to detect differences. Our study also benefitted from using data collected as part of routine prenatal care, which increases the consistency and accuracy. The data also included several confounders for which we were able to control. The JEM constructed to measure psychosocial work exposures had separate measures for men and women, which makes exposure measurements more applicable to the women in our study than general measures would. Finally, we were able to test whether many other occupational exposures were potential confounders, which has not been done in previous research. The JEM used for these tests, as well as for the main exposure definition, were developed by occupational health experts on Swedish working conditions. This is an important and unique strength of our study in that it provides a more objective measurement of exposure than could be obtained through self-report.

In conclusion, our results show an increased risk in HDP and GDM for women working in jobs with low levels of decision authority, but a protective effect for those working in jobs with high demands. Results remained the same for women who were in their first pregnancy and working full-time. We recommend that studies investigate whether improvements in psychosocial working conditions and increased control over work tasks for pregnant women can mitigate these risks.

## Supplementary material

Supplementary material
