# Supplementary material for: Occupational stress and pregnancy-related hypertension and diabetes: Results from a nationwide prospective cohort
Source: Scand J Work Environ Health. 2022 Mar 31;48(3):239–47. doi: 10.5271/sjweh.4004 (PMC9523467; doi:10.5271/sjweh.4004)
Supplement: Supplementary material [file SJWEH-48-239-S001.pdf]

Occupational stress and pregnancy-related hypertension and diabetes: Results from a nationwide prospective cohort[1]

by Claudia Lissåker, PhD,[2] Tomas Hemmingsson, PhD, Katarina Kjellberg, PhD, Petra Lindfors, PhD, Jenny Selander, PhD

1. *Supplementary material*
2. *Correspondence to: Claudia Lissåker, Unit of Occupational Medicine, Institute of Environmental Medicine, Karolinska Institutet, Box 210, SE-171 77, Stockholm, Sweden. [E-mail: claudia.lissaker@ki.se]*

Translated questions from the Swedish Work Environment Survey used to create each component of occupational stress.

Decision authority

Can you partially decide when work tasks should be done?
Do you have the opportunity to decide your own work pace?
Can you take short breaks to talk pretty much any time?
Are you ever involved in deciding how your work is organized?

Psychological demands

Are you sometimes so stressed that you do not have time to talk about or even think about something besides work?
Do you sometimes have so much to do that you have to work during lunch, work overtime, or take work home?
Does your work require all of your attention and concentration?

Social support

Are you able to receive support and encouragement from colleagues when work feels difficult?
Are you able to receive support and encouragement from managers when work feels difficult?
If tasks feel very difficult, are you able to receive advice or help?
Does your manager show appreciation for things you have done?
Do other people show appreciation for things you have done?
